# Refractory hypoglycaemia in a dog infected with *Trypanosoma congolense*

**DOI:** 10.1051/parasite/2016001

**Published:** 2016-01-21

**Authors:** Jack-Yves Deschamps, Marc Desquesnes, Laetitia Dorso, Sophie Ravel, Géraldine Bossard, Morgane Charbonneau, Annabelle Garand, Françoise A. Roux

**Affiliations:** 1 Emergency and Critical Care Unit, ONIRIS, School of Veterinary Medicine, La Chantrerie CS 40706 44307 Nantes Cedex 03 France; 2 Centre de Coopération Internationale en Recherche Agronomique pour le Développement (CIRAD), UMR Intertryp 34398 Montpellier France; 3 Faculty of Veterinary Medicine, Kasetsart University Chatuchak, Bangkok 10900 Thailand; 4 Institut de Recherche pour le Développement (IRD), UMR Intertryp, LRCT Campus International de Baillarguet 34000 Montpellier France

**Keywords:** *Trypanosoma congolense* forest-type, Dog, Hypoglycaemia, France

## Abstract

A 20 kg German shepherd dog was presented to a French veterinary teaching hospital for seizures and hyperthermia. The dog had returned 1 month previously from a six-month stay in Senegal and sub-Saharan Africa. Biochemistry and haematology showed severe hypoglycaemia (0.12 g/L), anaemia and thrombocytopenia. Despite administration of large amounts of glucose (30 mL of 30% glucose IV and 10 mL of 70% sucrose by gavage tube hourly), 26 consecutive blood glucose measurements were below 0.25 g/L (except one). Routine cytological examination of blood smears revealed numerous free extracytoplasmic protozoa consistent with *Trypanosoma congolense*. PCR confirmed a *Trypanosoma congolense* forest-type infection. Treatment consisted of six injections of pentamidine at 48-hour intervals. Trypanosomes had disappeared from the blood smears four days following the first injection. Clinical improvement was correlated with the normalization of laboratory values. The infection relapsed twice and the dog was treated again; clinical signs and parasites disappeared and the dog was considered cured; however, 6 years after this incident, serological examination by ELISA *T. congolense* was positive. The status of this dog (infected or non-infected) remains unclear. Hypoglycaemia was the most notable clinical feature in this case. It was spectacular in its severity and in its refractory nature; glucose administration seemed only to feed the trypanosomes, indicating that treatment of hypoglycaemia may in fact have been detrimental.

## Abstract (fr)

Un chien Berger allemand de 20 kg a été présenté à un hôpital universitaire vétérinaire français pour convulsions et hyperthermie. Le chien était revenu un mois auparavant d’un séjour de 6 mois au Sénégal et en Afrique sub-saharienne. La biochimie et l’hématologie ont montré une hypoglycémie sévère (0.12 g/L), une anémie et une thrombocytopénie. Malgré l’administration de grandes quantités de glucose (30 mL de glucose 30 % par voie IV et 10 mL de sirop de sucre à 70 % par une sonde naso-oesophagienne, chaque heure) 26 mesures consécutives de la glycémie (sauf une) étaient inférieures à 0,25 g/L. Un examen cytologique de routine de frottis sanguins a révélé de nombreux protozoaires extracytoplasmiques libres compatibles avec *Trypanosoma congolense*. La PCR a confirmé une infection par *Trypanosoma congolense* de type forêt. Le traitement a consisté en 6 injections de pentamidine à 48 heures d’intervalles. Les trypanosomes ont disparu des frottis sanguins 4 jours après la première injection. L’amélioration clinique a été corrélée à la normalisation des valeurs de laboratoire. L’infection a rechuté 2 fois et l’animal a reçu de nouveaux traitements ; les signes cliniques et les parasites ont disparu et le chien a été considéré comme guéri ; toutefois, six ans après cet incident, le test ELISA pour *T. congolense* était positif. Le statut de ce chien (infecté ou non infecté) demeure incertain. L’hypoglycémie était la caractéristique clinique la plus notable de ce cas. Elle était spectaculaire dans sa sévérité et dans son caractère réfractaire ; l’administration de glucose ne semblait que nourrir les trypanosomes laissant à penser que le traitement de l’hypoglycémie avait pu en fait être néfaste.

## 1. Case presentation

### 1.1. Signalment – history

A 20 kg, 2-year-old intact female mixed-breed German shepherd dog was referred to the Emergency unit of the Nantes Veterinary Teaching Hospital (France) for several seizures and persistent hyperthermia. The dog had returned 1 month previously from a six-month stay in Senegal and sub-Saharan Africa. She had been properly vaccinated and dewormed. The rabies antibody titre was 7.92 IU/mL, above the value required for re-entry to Europe. The owners reported lethargy, reduced appetite, weight loss and fever peaks > 40 °C for 6 weeks. The dog had no history of epilepsy.

### 1.2. Physical examination

**Figure 1.**
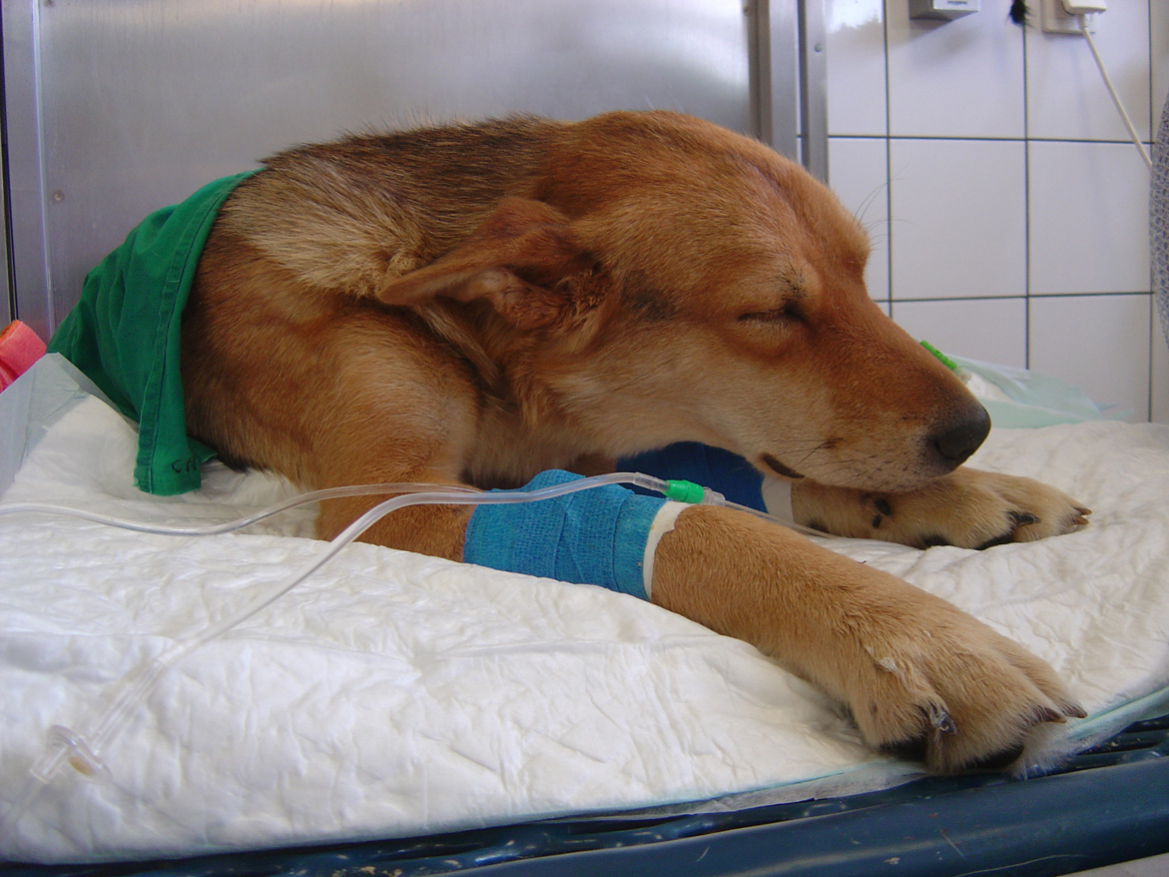
At presentation, the dog was cachectic, sleepy but responsive, sternally or laterally recumbent.

At presentation, the dog was cachectic, sleepy but responsive, sternally or laterally recumbent (Fig. 1), with hyperthermia (39.9 °C). Physical examination revealed pale mucous membranes, capillary refill less than 2 s, normal hydration, tachypnoea (60 breaths/min) and tachycardia (heart rate 140 bpm). There were no petechiae. The skin showed sequelae of myiasis (most probably due to *Cordylobia anthropophaga*), but no ulcers. A tick (female *Rhipicephalus sanguineus*) was identified on the coat. Superficial lymph nodes were enlarged, especially the popliteal lymph nodes. There was no ocular anomaly.

### 1.3. Hypothesis

Seizures led to suspicion of a metabolic disorder, such as hypoglycaemia. Encephalitis was also suspected; rabies was considered. Epilepsy was excluded because of the associated symptoms. Poisoning was excluded because of the duration of symptoms. Fever and lymph node enlargement led to increased suspicion of systemic infectious diseases like leishmaniasis, ehrlichiosis, borreliosis, anaplasmosis or babesiosis. Due to the travel history, infection by an indigenous African infectious agent was considered. Lymphoma was not excluded.

### 1.4. Analysis

Routine biochemistry and haematology showed severe hypoglycaemia (0.12 g/L, reference 0.6–1.1 g/L), normocytic (mean corpuscular volume 72 fl; reference interval 61–77 fl), normochromic (mean corpuscular haemoglobin concentration 33 g/dL; reference interval 33–36 g/dL), hyporegenerative (reticulocytes 49,600/μL; reference interval 0–60,000/μL), anaemia (haemoglobinaemia: 58 g/L, reference 120–180; erythrocytes: 2.6 × 1012/L, reference 5.5–8.5 × 1012/L) and thrombocytopenia (45 × 10^9^/L, reference 200–500 × 10^9^/L). Blood type was DEA 1.1+. Other abnormalities included hypoalbuminaemia (23 g/L; reference interval 25–35 g/L), hyperglobulinaemia (59 g/L; reference interval 30–45 g/L) and hypokalaemia (3.3 mmol/L; reference interval 3.8–5.2 mmol/L). Lactate, blood gas, alkaline phosphatase, alanine aminotransferase, bilirubin, urea, creatinine, prothrombin time and activated partial thromboplastin time were within reference ranges. Urinalysis was unremarkable. Systolic, diastolic and mean arterial blood pressures were within normal values. A blood smear was prepared at the time of blood sample collection and stained with Wright-Giemsa (Diff Quick). The lymph nodes were aspirated and stained.

### 1.5. Diagnosis

**Figure 2.**
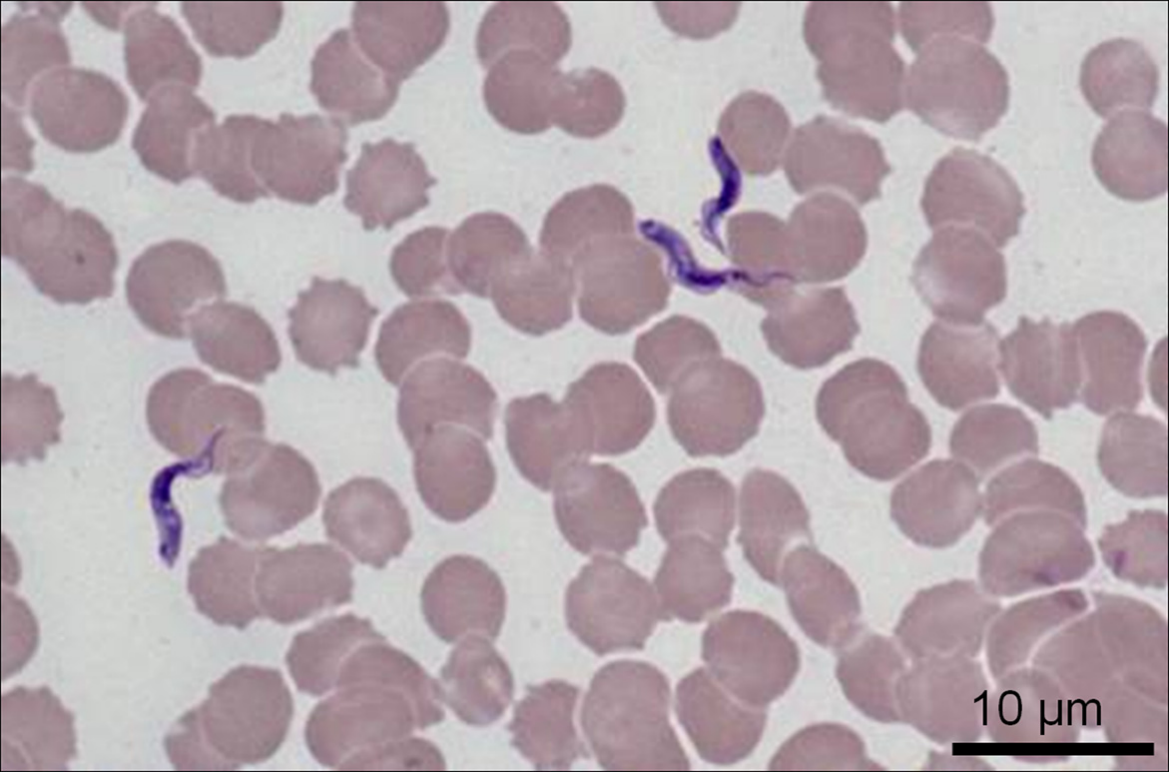
Cytological examination of blood smears showed numerous free parasites (May-Grünwald-Giemsa stain).

**Figure 3.**
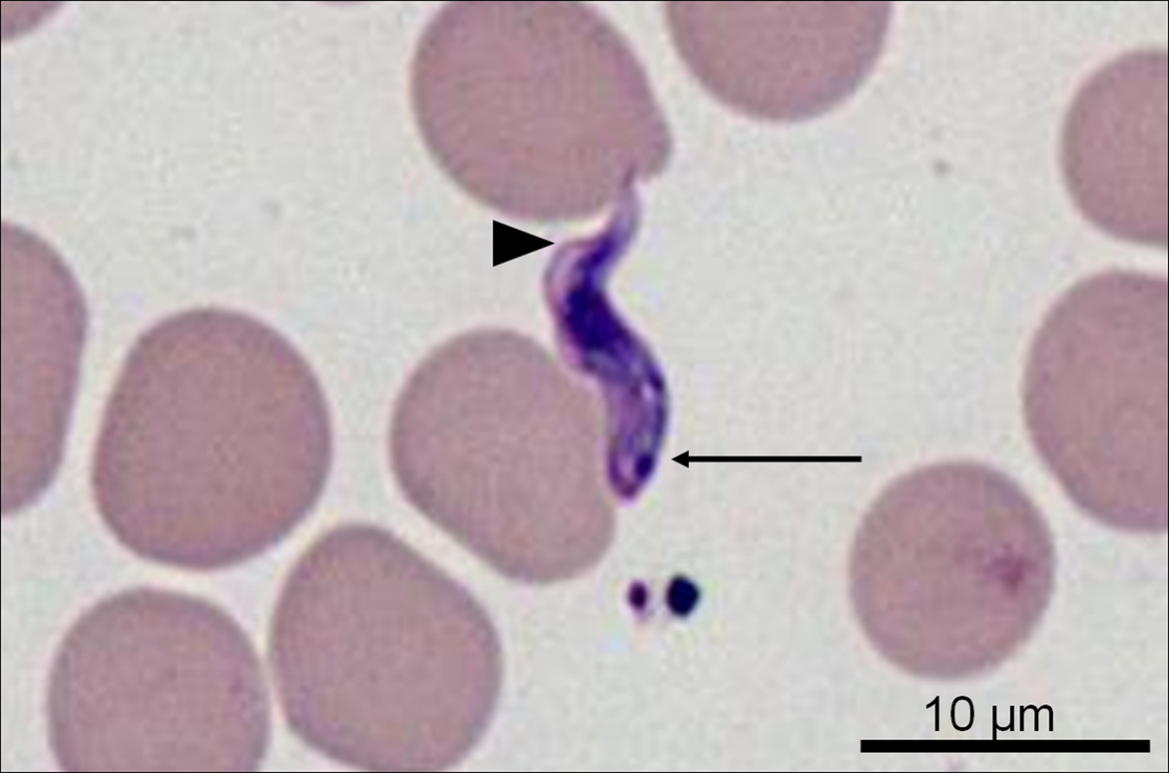
The protozoa are 10–20 μm in length and characterized by a central nucleus, a medium-sized kinetoplast located at the margin of the body, in a sub-terminal position (arrow), a poorly-developed undulating membrane (arrowhead) and no free flagellum (May-Grünwald-Giemsa stain).

Routine cytological examination of the blood smears revealed numerous free extracytoplasmic protozoa (Fig. 2). The parasites were 10–20 μm in length, 3 μm wide, with a central nucleus, a medium-sized kinetoplast located at the body margin, just in front of the posterior extremity (marginal and sub-terminal kinetoplast), a poorly-developed undulating membrane and no free flagellum (Fig. 3). This morphology is consistent with *Trypanosoma congolense* [9]. The blood smears showed no *Babesia canis*. Numerous trypanosomes were also present in lymph node aspiration. These observations pointed to a diagnosis of canine African trypanosomosis. *Babesia* and *Leishmania* serologies were negative at all dilutions. Polymerase chain reaction (PCR) confirmed a *Trypanosoma congolense* forest-type infection. All the other taxa-specific PCRs were negative (*Borrelia*, *Ehrlichia* and *Leishmania*).

### 1.6. Staff protection

*Trypanosoma congolense* does not infect humans and the vector, the tsetse fly, is not present in France. No preventive measures were taken for the medical staff, except, as for any patient, not to handle biological fluids without gloves.

### 1.7. Critical care

The dog was transferred to the intensive care unit (ICU). On admission to the ICU, two catheters were placed, one for fluid administration and drug injection, the other for blood collection. A naso-oesophageal tube was placed for sucrose administration.

#### 1.7.1. Anaemia and thrombocytopenia management

Anaemia, thrombocytopenia, tachypnoea and tachycardia led to transfusion of 10 mL/kg fresh whole blood. Mucous membranes became more pink, heart rate and respiratory rates returned to normal values and biological parameters improved (haemoglobinaemia: 79 g/L; red blood cells: 3.6 × 10^12^/L; thrombocytes: 135 × 10^9^/L), but the dog remained in lateral or sternal recumbency, unable to get up, sleepy, almost slumbering.

#### 1.7.2. Hyperthermia management

**Figure 4.**
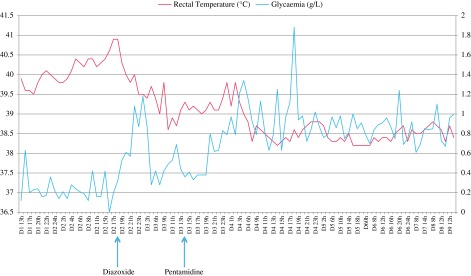
Temperature and glycaemia as a function of time.

On day 1, at 1 PM, the body temperature was 39.9 °C; at 2 PM 40.0 °C; at 3 PM 39.8 °C; and at 4 PM 39.6 °C. Practical cooling methods were instituted: a fan, moist towel, blocks of ice and alcohol wipes. They had no effect (Fig. 4).

#### 1.7.3. Hypoglycaemia management

Hypoglycaemia was the major concern.

On day 1, at 1 PM, glycaemia was only 0.12 g/L and the dog had a new seizure. The first measurements and most of the very low measurements were carried out simultaneously with a glucometer and a calibrated laboratory analyser (Idexx^®^); no discrepancy was found between them. The dog was placed on a 5% glucose solution with 20 mmol/L KCl at a rate of 1 mL/kg/h, except during transfusion. The severe hypoglycaemia was treated with a 30 mL of 30% glucose IV bolus injected slowly at the injection site of the maintenance solution to prevent phlebitis. Ten millilitres of sucrose 70% (7 g/10 mL), Canadou^®^, a sugar syrup used for sweetening cocktails, was administered by gavage tube.

At 4 PM, glycaemia was 0.63 g/L. At 5 PM, glycaemia was 0.2 g/L. From 6 PM until 3 PM on day 2, the dog had glycaemia measurements every hour, i.e. 26 measurements, all below 0.25 g/L, with the exception of one of 0.36 g/L at 11 PM (Fig. 4). After each measurement, the dog received the same supplements of glucose and water, i.e. 10 mL of sucrose 70% by gavage tube, 30 mL of glucose 30% IV through the tubing of the CRI of 1 mL/kg of 5% glucose, and Hill’s a/d^®^ food given by hand, for a total of about 20 g of glucose/hour (1 g/kg/h) and 60 mL of fluids/hour (3 mL/kg/h). The dose of glucose administered was approximately two times that usually recommended. Despite this support, no clinical or physiological improvement was seen. Despite the lack of effect on glycaemia, glucose supplementation was continued to prevent the irreversible or even fatal neurological effects associated with profound hypoglycaemia. At midnight, the dog presented with head-shaking, the glycaemia then being 0.22 g/L. At 4 PM, glycaemia was undetectable (“value too low”) with the glucometer (the detection limit was 0.1 g/L).

At 6 PM on day 2, the dog received diazoxide, Proglycem^®^, at a dose of 2.5 mg/kg, a glucose-elevating drug used to treat refractory hypoglycaemia induced by insulinoma. Glycaemia increased. Glycaemia was 0.2 g/L at 5 PM, 0.32 g/L at 6 PM, 0.53 g/L at 7 PM, 0.61 g/L at 8 PM, 0.57 g/L at 9 PM and 1.08 g/L at 10 PM. At that time, the dog was standing upright and was able to walk. Supplemental glucose was then limited to a maintenance solution of 5% glucose at 1 mL/kg/h supplemented with K+ and 10 mL of 70% sucrose by gavage tube. The next four readings were nearly normal: 0.87 g/L at 11 PM, 1.18 g/L at 0:00 on day 3, 0.87 g/L at 2 AM, but by 4 AM glycaemia had dropped to 0.28 g/L. The initial treatment every hour and diazoxide every 12 h were insufficient to restore normoglycaemia, with values remaining around 0.4 g/L all of the third day.

Every hour, after glycaemia and body temperature measurements, the dog’s position was shifted in order to prevent pressure ulcers. Arterial blood pressures, pulse and respiration rates taken every hour were always within normal limits.

### 1.8. Specific treatment

Animal trypanocides are not available in metropolitan France; as a result, a human product was used. The only drug quickly available was pentamidine, Pentacarinate^®^. It was ordered, and arrived on day 3. Treatment consisted of six injections at 4 mg/kg at 48-hour intervals [1]. On day 3, at 2 PM, the dog received an infusion of 80 mg pentamidine diluted in 50 mL of 5% glucose (normal saline is contraindicated, according to the manufacturer) over a period of 4 h in order to watch for signs of anaphylaxis. No adverse effects were noticed but hepatic parameters increased (ALP: 388, reference 0–200 IU/L, ALT: 201, reference 0–80 IU/L). The next five doses were administered by intramuscular injection every 48 h. Glycaemia remained low and temperature high until 1 AM on day 4 before normalizing (Fig. 4). Clinical improvement was correlated with the normalization of laboratory values. Trypanosomes had disappeared from the blood smears four days following the first injection. On day 14, the day following the last injection, the dog was discharged.

### 1.9. Relapses

Two weeks after discharge, or 1 month after initial presentation, the dog again presented with the same symptoms, an enlarged spleen and bilateral corneal opacity. Routine chemistry and haematology revealed anaemia (haemoglobinaemia: 66 g/L, erythrocytes: 2.8 × 10^12^/L), hypoglycaemia (0.68 g/L) and thrombocytopenia (42 × 10^9^/L). Many trypanosomes were present on the blood smears. Ultrasound revealed marked splenomegaly with multinodular parenchyma and a hypoechoic pattern, consistent with reactive splenitis. A second transfusion was performed. Thirteen glycaemia measurements during the first 24 h were all low, with a minimum of 0.31 g/L and a maximum of 0.59 g/L (Fig. 4). Hypoglycaemia was treated as in the first episode, with no greater success. The dog received three other intramuscular injections of 4 mg/kg pentamidine at 72-hour intervals.

Three weeks after the first relapse, or 7 weeks after the first admission, the dog again presented, after a seizure. Haematology still showed anaemia (erythrocytes: 4 × 10^12^/L) and thrombocytopenia (120 × 10^9^/L). Again, parasites were visible on the blood smears. A third transfusion was performed. Again, three intramuscular injections of 4 mg/kg pentamidine were given, but at 10-day intervals. The dog came back for monthly examinations for 3 months, upon which clinical examinations, biochemistry and haematology were within normal ranges.

Three months, 6 months and 1 year after the last episode, no trypanosomes were visible on the smears. The dog even became pregnant 1 year later and gave birth to healthy puppies. She was considered cured.

Six years after this incident the dog was presented for a physical examination prior to another trip to Senegal; she was healthy, no parasites were found on a blood smear, but serological examination by ELISA *T. congolense* (using soluble antigens of *T. congolense* Savannah-type) was positive. PCR was negative.

## 2. Discussion

### 2.1. Canine trypanosomosis

There are six *Trypanosoma* species or types described in carnivores worldwide [8]. *T. cruzi*, responsible for American trypanosomosis or Chagas disease, is not present in Africa. *T. evansi* is a trypanosome mainly found in camels in Senegal. Dogs are rarely found to be infected with *T. vivax*, although there have been some reports in Cameroon [26]. The major African trypanosomoses affecting dogs are *T. congolense* and two subspecies of *T. brucei*: *T. brucei brucei* and *T. b. gambiense* [11, 40]. In the present case, *T. brucei* and *T. evansi* were ruled out based on morphologic criteria.

Three types of *T. congolense* are described (from the most pathogenic to the least): *T. congolense* savannah-type is most often found in cattle, in which it produces a rapidly fatal or severe infection, whereas *T. congolense* forest-type causes more chronic infection in cattle, but also in dogs. *T. congolense* kilifi-type is rarely reported, since it usually causes an asymptomatic infection [3]. These parasites are quite closely related and serological responses show strong cross-reactions between the three parasites; thus both homologous and heterologous ELISAs can be used to detect serological responses to these three parasites [6].

**Figure 5.**
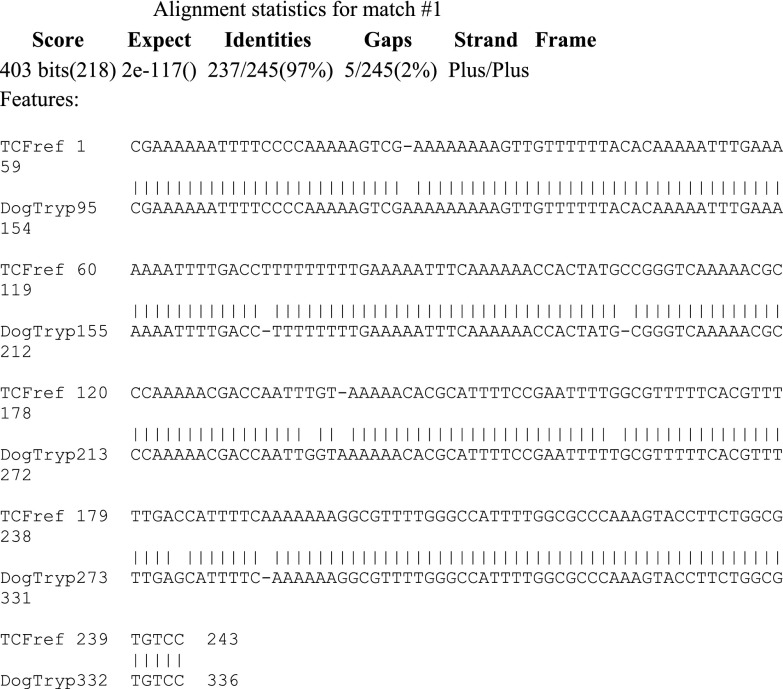
Comparison of the dog’s trypanosome sequence with the reference sequence published by Masiga et al. [21].

In the present case, a *Nannomonas Trypanosoma* sp. was characterized by microscopic observation of the parasites; a definitive diagnosis of *T. congolense* forest-type was reached using PCR sequencing confirmation (Fig. 5) [8].

African animal trypanosomosis is called *nagana* in tropical Africa. Trypanosomes are transmitted by tsetse flies of the genus *Glossina*, widespread in this region [14]. The parasites are ingested by the tsetse flies during a blood meal, where they begin a development cycle, including multiplication in the vector; they may then be inoculated into another mammalian host. Canine trypanosomosis caused by *T. congolense* is non-zoonotic, but there is a human equivalent of *nagana*, human African trypanosomosis, also known as sleeping sickness, whose agents are *Trypanosoma brucei gambiense* and *T. b. rhodesiense*, the vector of which is also the tsetse fly.

Canine African trypanosomosis is less common than bovine trypanosomosis, a major constraint to livestock productivity; however, in endemic countries, the observed prevalence in dogs can reach 30.1% [18]. In endemic countries, European dogs die soon after trypanosomal infection, while African dogs develop milder parasitaemias and may remain clinically asymptomatic, possibly because of trypanotolerance [17]. Cases reported outside the endemic areas of Africa are all imported.

To our knowledge, there are only five previous case reports in Europe of canine African trypanosomosis in dogs other than military.

The first case, published in a non-indexed journal, presented in France, was a German shepherd dog returning from Senegal infected with *Trypanosoma congolense* [10]. He presented with lethargy, anorexia and hyperthermia. He recovered.

The second case, presented in Scotland, was a Jack Russell Terrier returning from Mozambique infected with *Trypanosoma congolense* savannah-type [13]. He presented with abdominal distension, anaemia and thrombocytopenia, and died shortly after admission. It must be emphasized that this animal spent 3.5 years in the UK before the infection was detected.

The third case, presented in the Netherlands, was a Vizla returning from Senegal, that presented with anaemia; the *Trypanosoma* species was not identified [33]. The dog was treated with pentamidine and recovered.

The fourth case was in France, a German wire-haired pointer returning from Senegal infected with *Trypanosoma congolense* savannah-type [23]. He presented with anaemia and thrombocytopenia, and died 2 weeks later.

The fifth case was also in France, in a Shih Tzu returning from Senegal infected with atypical hyperpachymorph *Trypanosoma congolense* forest-type [9]. He presented with anaemia and died four days after admission.

Besides these dogs owned by individuals, canine trypanosomosis is regularly observed in France in military working dogs returning from sub-Saharan African countries [5, 18, 37].

### 2.2. Hypoglycaemia

Hypoglycaemia is the most notable clinical feature of this case. Hypoglycaemia is part of the classical presentation of trypanosomosis; it has been reported in several species, as in buffaloes [19], in buffalo calves [20], in goats [24], in camels [27], in a tiger [34], in a dog [15] and in a human [25].

Hypoglycaemia explains the lethargy and convulsions. The fact that the dog was able to stand and walk when her blood sugar rose to 1.08 g/L (at 10 PM on day 2) reinforces the impression that symptoms were due mainly to hypoglycaemia.

Hypoglycaemia was spectacular in its severity: most values were <0.4 g/L and often close to 0.2 g/L, which in general is not compatible with life. However, the clinical effects were less severe than the laboratory values would suggest. At 4 PM on day 1, blood glucose was undetectable, yet the dog was not comatose or convulsing.

The most striking observation was its refractory nature. There was a total lack of response to glucose administration. Severe hyperglycaemias are sometimes reported, but intravenous or oral glucose administration always causes an increase in glycaemia, at least temporary. Every hour, this dog received 7 g of oral sucrose, 10 g of IV glucose and rich food, for a total of almost 20 g of sugar every hour (1 g/kg/h), or 500 g of pure sugar per day. In total, over the four days that hypoglycaemia lasted, the dog received 2 kg of pure sugar (equivalent to two boxes of sugar), without any effect. The same phenomenon happened again during the first relapse, when the dog received almost 1 kg of pure sugar in two days. To our knowledge, such severe hypoglycaemia in the context of such aggressive glucose supplementation has never been reported in the veterinary literature.

The origin of this severe and refractory hypoglycaemia was a crucial diagnostic question. Sepsis-induced hypoglycaemia is usually less pronounced, and not refractory. The hypothesis of rebound hypoglycaemia was rejected, because we never observed hyperglycaemic peaks. We did not measure insulin levels. Severe hypoglycaemia and hyperinsulinaemia have been observed in human patients infected with *Plasmodium falciparum*, attributed in part to large glucose requirements of malaria parasites [38]. In our patient, as soon as trypanosomes disappeared from the blood smears, glycaemia and temperature normalized. Trypanosomes appeared to be directly responsible for hypoglycaemia and hyperthermia. It was as if glucose administered to the dog only served to feed the trypanosomes, indicating that treatment of hypoglycaemia may have been detrimental.

We found old studies in the 1930s that seem to support this hypothesis. Trypanosomes use enormous amounts of sugar for their metabolism; they consume twice their own mass of sugar within 24 h [39]. Insulin injection into dogs or rats infected experimentally leads to a reduction in the number of trypanosomes [29, 30]. In infected rats, hyperglycaemia after injection of glucose increased the population of trypanosomes [4, 28]. Trypanosome mobility decreased in blood samples at room temperature, but moribund trypanosomes could be revived by the addition of sugar [31]. In 1937, hypoglycaemic therapy was considered for the management of trypanosomosis [2, 31, 36], but this line of study was abandoned with the arrival of trypanocides [32].

These old experimental studies were reinforced by two clinical observations in human patients.

In a case reported in the United States to CDC internal records, a diabetic patient with African trypanosomosis required less insulin during the acute phase of his illness (Ackley A., personal communication, quoted by Nieman [12, 25]).

Another case observed in the United States described severe African trypanosomosis with spurious hypoglycaemia in a 58-year-old woman returning from Kenya and Rwanda [25]. Despite severe hypoglycaemia on admission (0.21 mg/L), the patient was fully alert, with no clinical manifestation of hypoglycaemia. Despite the IV injection of hypertonic glucose solutions, blood glucose levels continued to register as <0.50 mg/L. The authors considered the possibility of spurious hypoglycaemia due to *in vitro* utilization of glucose by trypanosomes. They drew two blood samples simultaneously. The first sample was placed on ice and carried to the laboratory; the glucose level was 1.03 mg/L. In the second sample, not placed on ice and analysed at the same time, the glucose level was only 0.48 mg/L. At the same time of venepuncture, in a capillary blood specimen, the blood glucose level, given by a rapid assay method (Accucheck^®^), was 1.07 mg/L. In our case, most measurements were performed with a glucometer, immediately after the sample was drawn, or with an analyser located in the ICU, so the possibility of spurious hypoglycaemia is improbable. However, this case emphasizes the large consumption of glucose by trypanosomes, even *ex vivo*.

### 2.3. Specific treatment and follow-up

There are no trypanocides currently available in metropolitan France although some of them may be found. Melarsomine dihydrochloride, sold for the control of heart worm under the registered name Immiticide, is a trypanocide also sold in Africa under the registered name Cymelarsan for the control of surra (sub-genus *Trypanozoon*) in camels; it is not known as an active trypanocide against the sub-genus *Nanomonnas*, so it would not be suitable for the present case. Nifurtimox, a human drug, may be found in hospitals but rather targets *Trypanosoma cruzi*, and was not fully validated against *Trypanozoon* parasites; moreover, it may not be well suited for animal treatments. Pentamidine was discovered accidentally in 1937 in the course of research on blood sugar lowering agents which have the ability to disrupt the parasite’s energy metabolism [32]. Pentamidine is used in humans for the control of *T. brucei* ssp. (Trypanozoon). It was not evaluated against *T. congolense* in animals, and as a result, there are no data available on its efficacy against *T. congolense* or on resistance. Diminazene aceturate is not available in France and is thought to be toxic in dogs. However, it is currently used in dogs in Africa and Asia respectively against *T. congolense* and *T. evansi* [7]. Pentamidine was shown to be more efficient than diminazene (Berenil^®^), the standard veterinary trypanocide, in the treatment of *T. brucei brucei* infection in dogs [1]. Isometamidium (Trypamidium^®^) requires only a single intramuscular injection at 1 mg/kg, but is only available in Africa. Isometamidium is used every 2 months for chemoprophylaxis in military dogs in sub-Saharan Africa [37].

This is the third non-fatal case of canine African trypanosomosis reported [10, 33].

Two relapses were observed. Failures in trypanocide treatments originate from (i) an extravascular refuge of the parasite (which generally induces late or very late relapses), (ii) insufficient treatment (underdosage, incorrect route of administration, contamination, counterfeit product, etc.) or (iii) parasite chemoresistance. In the last two cases, the relapse generally occurs within 2–4 weeks, due to the fact that some of the parasites have not been killed. In our study, the treatment procedure was carefully applied and is considered to be reliable in terms of trypanocide quality, quantity and administration route. In this case, after a first series of six pentamidine administrations, the very early relapse observed is suggestive of parasite chemoresistance to pentamidine. Indeed, the second series of treatments was also followed by a very early relapse, which confirms the fact that some of the circulating parasites were not killed by the trypanocide treatment. More problematic is the fact that the parasite disappeared after the third treatment at the same dose, but applied at 10-day intervals. It is hypothesized that repeated treatments, that proved to be sub-curative, may have enhanced the immune system and have finally led to either a self-cure or a clinical cure with sustainable control of the parasitaemia down to undetectable levels. Iterative treatments that proved to be sub-curative would then be a way to achieve a curative treatment protocol. However, such situations reveal pre-existing drug resistance and would even increase the selection of more resistant strains which would be another important concern in enzootic countries.

The actual status of the dog in our case is questionable. There are no specific data available on the persistence of antibodies to trypanosomes in dogs. However, based on the observations made in livestock [35], persistence of antibodies 6 years after a cure is not expected. Seropositivity 6 years after this episode is rather suggestive of healthy carrying of the parasite. This hypothesis could not be confirmed by PCR based on a blood sample collected in November 2015, since it was negative. At this stage, laboratory results suggest that the dog is still infected, but remain unable to confirm infection. Due to this uncertain status, it is highly recommended to administer a new treatment to the dog, in order to eliminate potential carrying. Quinapyramine salts (sulfate and chloride) at 5 mg/kg is suggested. Later on, the animal should be followed up using ELISA *T. congolense* (and PCR). A successful curative treatment would most probably lead to negative seroconversion within 6 months post-treatment, as is usually observed with African trypanosomosis in other animals.

In the present case, the risk of transmission to humans was low, since (i) *T. congolense* does not affect humans; and (ii) the parasite requires a vector not present in France. However, dogs can be infected with *T. brucei* that infect humans and mechanical infection can occur in areas where tsetse flies are not present. Quarantine after return from endemic countries is sometimes recommended [16]. Quarantine could however be insufficient because the incubation period can be very long. In the case reported by Gow et al., the asymptomatic period was 3.5 years [13]. Our dog might also be an asymptomatic carrier 6 years after her apparent cure. Dogs that return from endemic countries of human trypanosomiasis should be investigated by molecular screening as well as serological tools.

### 2.4. PCR strain identification

**Figure 6.**
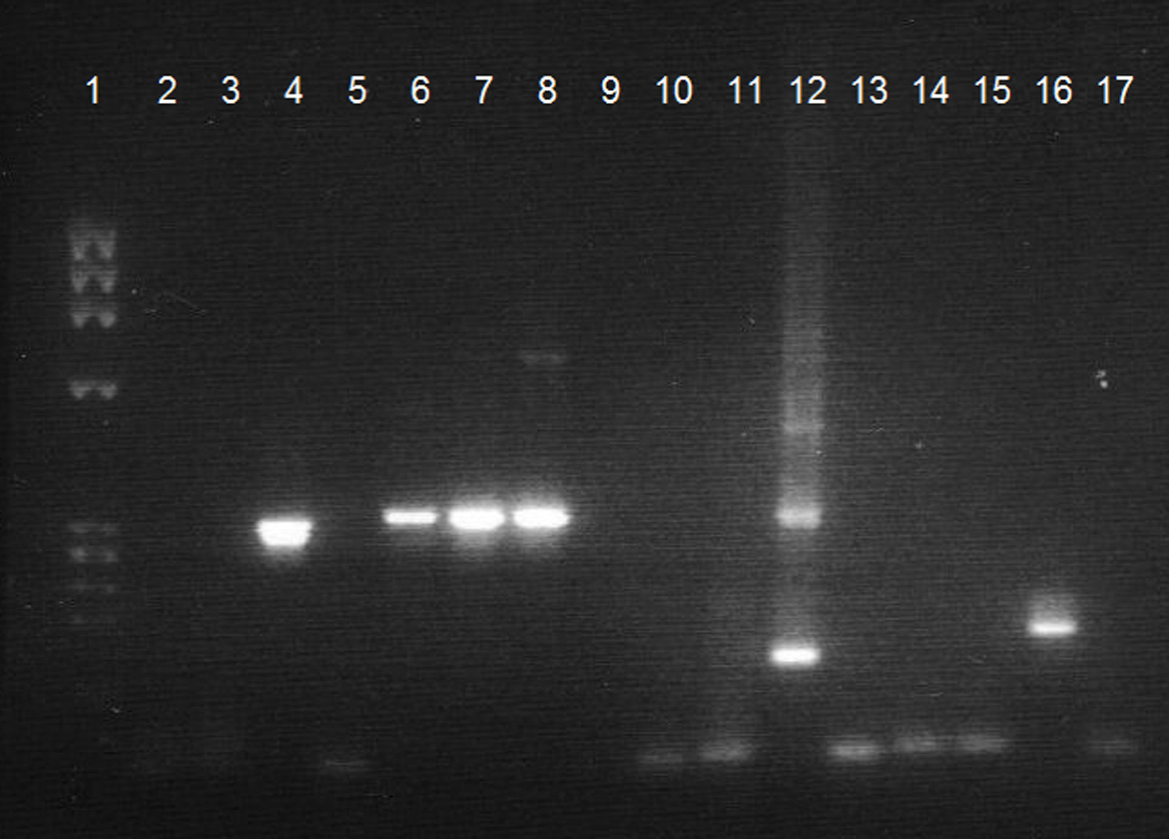
1: Molecular marker (PhiX174/HaeIII); 2, 3, 6, 7, 10, 11, 14, 15: blood of the dog; 4: *T. congolense* savannah-type positive control; 8: *T. congolense* forest-type positive control; 12: *T. vivax* positive control; 16: *T. brucei* positive control; 5, 9, 13, 17: negative controls.

The virulence of *Trypanosoma congolense* varies greatly with type. Therefore, PCR analysis of whole blood was performed to correctly identify this *Trypanosoma* sp. and type. The sensitivity of PCR is two to three times higher than microscopic observation of the buffy coat, and the sensitivity threshold of trypanosome detection generally ranges from 1 to 20 parasites/mL of blood. For monospecific PCR identification, the most favoured target is minichromosomal nuclear satellite DNA, which has the advantage of a highly repetitive short sequence (10,000–20,000 repeats; 120–600 bp). Specific satellite primers were used for *Trypanozoon*, *T. vivax*, *T. congolense* savannah-type, *T. congolense* forest-type [21, 22]. The resulting PCR amplification product of 350 bp, expected with *T. congolense* forest-type infection using TCF1/TCF2 primers, can be visualized on an agarose gel after staining with ethidium bromide and exposure under ultraviolet light (Fig. 6). The specific size of the PCR product was evaluated by simultaneous migration of molecular size markers and of a positive control [8]. The sequence of the amplification product shows 97% identity with the published reference sequence of Masiga et al. [21] (Fig. 5). Additionally, Pan-trypanosome primer sets were used for *Trypanosoma* spp. detection: TRYP4 and nested PCR targeting the 18S-ITS1 region, confirming the presence of *T. congolense* forest-type (data not shown).

## 3. Conclusion

In this dog, infection with *Trypanosoma congolense* forest-type caused severe hypoglycaemia refractory to sugar supplementation. It is possible that the drastic treatment of hypoglycaemia had the paradoxical effect of promoting trypanosome survival. The dog survived but is suspected of being an asymptomatic carrier 6 years after her apparent cure. Further treatment and follow-up should help to clarify and hopefully solve this unclear status.
